# Cardiac Doppler Parameters and Progress in Clinical Manifestation of Primary Lower Extremity Varicose Veins: A Prospective Study in China

**DOI:** 10.3389/fsurg.2022.791598

**Published:** 2022-02-28

**Authors:** Jia Zhang, Zhoupeng Wu, Qingbo Feng, He Huang, Yukui Ma

**Affiliations:** ^1^West China Clinical Medical College, West China Hospital of Sichuan University, Chengdu, China; ^2^Department of Vascular Surgery, West China Hospital of Sichuan University, Chengdu, China; ^3^Department of Liver Surgery and Liver Transplantation Centre, West China Hospital of Sichuan University, Chengdu, China; ^4^Department of Cardiology, West China Hospital of Sichuan University, Chengdu, China

**Keywords:** primary varicose veins, cardiac Doppler, cardiac haemodynamics, CEAP, age

## Abstract

**Objective:**

To investigate the features of cardiac Doppler parameters in patients with primary lower extremity varicose veins in China.

**Materials and Methods:**

We performed a prospective statistical analysis of cardiac Doppler parameters between 129 Chinese patients with varicose veins and normal controls. Furthermore, we evaluated the relationship between cardiac Doppler and the progress or severity of lower extremity varicose veins.

**Results:**

Compared with normal controls, patients with primary varicose veins had significantly lower early mitral and tricuspid diastolic inflow and annular velocities (E- and e′-waves), significantly higher late mitral and tricuspid diastolic inflow and annular velocities (A- and a′-waves), significantly higher mitral systolic annular velocities (s′-wave), and significantly lower mitral and tricuspid E/A ratio. There was no significant association between deep venous reflux (DVR) of the lower extremities and cardiac Doppler parameters. The relationship between Clinical Etiological Anatomical Pathophysiological (CEAP) clinical class and cardiac Doppler parameters showed on that: In comparison with normal control, all cardiac Doppler parameters of C2 clinical class patients were basically unchanged, but the cardiac Doppler parameters of the C3 or higher CEAP class patients changed. Hence, we found a potential CEAP grade cut-off value (C3) linked to statistical changes in cardiac Doppler parameters.

**Conclusion:**

Cardiac Doppler parameters in patients with primary varicose veins could indeed be different from those of normal people, especially for C3 class or higher CEAP clinical class patients. Therefore, for those patients, pre-operative echocardiography can be used to evaluate cardiac hemodynamic changes, but large-scale clinical promotion requires further large sample studies.

## Introduction

The prevalence of primary varicose veins has been reported to be as high as 60% in the adult population (1). The influence of varicose veins is reflected in the physical, mental, and social health of the individual. Varicose veins of lower limbs may be caused by non-functional superficial or deep venous valves, subsequent venous reflux, and rising peripheral venous pressure (2, 3). Other than for venous valves, venous return is also closely related to the central pump (heart) and the pressure gradients between the systemic capillaries and right ventricle (3–6). The aetiology, pathogenesis, pathophysiology, and clinical manifestations of primary varicose veins have been extensively studied in the peripheral venous system (2, 7–10). However, the relationship between varicose veins and cardiac haemodynamics is indistinct due to lack of research. In addition, the relationship between the progress or severity of primary varicose veins and cardiac haemodynamics also remains unknown. We conducted a prospective investigation of the features of cardiac Doppler parameters in Chinese patients with primary varicose veins. Based on this, we supposed whether there were some connexions between cardiac Doppler parameters and the severity in patients with varicose veins. Further, we explored the relationship between cardiac Doppler haemodynamics and the progress or severity in those patients.

## Materials and Methods

### Progress in Clinical Manifestations

The Clinical Etiological Anatomical Pathophysiological (CEAP) classification, published in 1994, is a widely adopted classification system for clinical application, papers, and reports on primary varicose veins (7). According to clinical manifestations, CEAP was divided into 7 grades, from C0 to C6. The increase of C grade indicated the progress of clinical manifestation. C0–C1 patients refer to those with telangiectasia or reticular veins and physical therapies, such as stretch socks or exercise, which are usually adopted. There is no adequate reason to persuade C0–C1 patients to undergo echocardiography that is not directly related to their diagnosis and treatment. Therefore, only C2–C6 patients who require surgery due to obvious clinical symptoms are included in our study.

C2 CEAP clinical class means true varicose veins, while C3 represents edoema due to venous disease. We merged C5 and C6 CEAP clinical class patients with C4 to generate a C-h group representing patients that had lesions in the surrounding area of varicose veins, e.g., pigmentation, eczema, lipodermatosclerosis, and varicose ulcer. Other than CEAP, we also analysed the relationship between age and gender and cardiac Doppler in varicose vein patients.

### Deep Vein Reflux

The severity of varicose veins is not only manifested as visual varicose veins and the deterioration in the lower limb skin. Internal manifestations, such as deep venous valve insufficiency and reflux (DVR), are also important factors that cause the occurrence of varicose veins. Furthermore, there is some connexion between DVR and the haemodynamics of the venous system. All included patients underwent the pre-operative lower extremity Doppler ultrasonography to assess deep vein status. Based on this, we further analysed the relationship between different degrees of DVR and cardiac Doppler parameters.

### Patient Preparation

**Figure 1:**
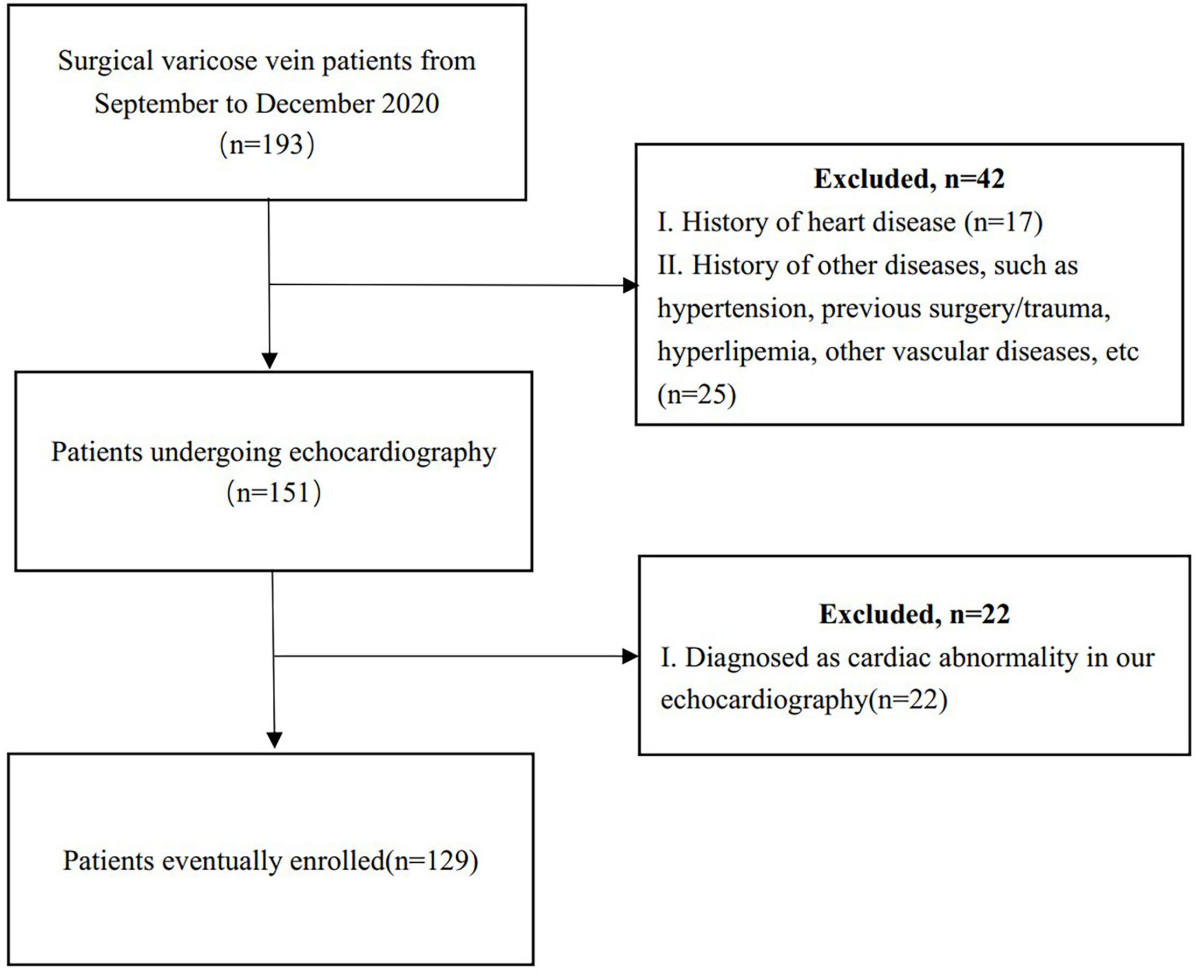
The inclusion and exclusion flow chart of patients with primary lower extremity varicose veins.

The C2–C6 primary lower extremity varicose veins patients without hypertension, coronary artery disease, valvular stenosis or calcification, pericardial constriction, deep vein thrombosis, and other conditions that have been verified to affect cardiac haemodynamics were included. Those that were previously undiagnosed but were evaluated to be abnormal cardiac structures and ventricular wall movements in our echocardiographic examinations were excluded. Echocardiographic Doppler, the condition of DVR, and clinical data of 129 enrolled pre-operative Chinese patients (age: 23–80 years) were prospectively collected from September to December 2020 (Figure 1). Fifty-seven patients had unilateral varicose veins of the lower extremity and were scheduled to undergo unilateral varicose vein stripping, while 72 patients had bilateral varicose veins to whom bilateral stripping was proposed. All patients signed informed consent forms. Fifty-seven percent were women. Sixteen patients did not have DVR, while 113 had DVR. In terms of CEAP classification, 51 patients (40%) were in the C2 clinical class, 28 (22%) were in C3, and 50 (38%) were in C-h (C4+5+6).

### Selection of Control

The EMINCA (echocardiographic measurements in normal Chinese adults) nationwide study, conducted by the Ultrasound Medicine Branch of the Chinese Medical Association, issued normal reference values of cardiac Doppler echocardiography for Chinese adults in 2016 (11). Their inclusion criteria required normal blood pressure without abnormal cardiac structure and function, consistent with our criteria. Hence, normal control in our study was derived from the EMINCA study.

### Doppler Examination

Before pre-operative echocardiography, all patients were required to remain quiet for 30 min to control heart rate and blood pressure within the normal range. According to the ASE (American Society of Echocardiography) guidelines, comprehensive echocardiography, including pulsed wave and tissue Doppler echocardiography, was executed to obtain cardiac Doppler parameters (24). In the apical four-chamber view of pulsed-wave Doppler, we measured mitral and tricuspid inflow velocities (E-, A-wave) to assess heart ventricle diastole and filling function (12). In the apical four-chamber view of the tissue Doppler, we obtained mitral and tricuspid annular velocities (e′-, a′-, and s′-wave) (13). For those parameters, E wave reflected atrioventricular pressure gradient (14) and was influenced by pre-load and ventricular diastolic function (15). The e′ wave was recognised as a reliable indicator of myocardial diastole (16) due to relative cardiac pre-load independence (17). The A and a′ wave represented atrial contraction, atrial fraction, and global atrial function (18, 19). Mitral and tricuspids′ wave could be used to assess ventricular contraction (20, 21). The E/A ratio indicated diastolic function, while the E/e′ ratio represented ventricular filling pressure and end-diastolic pressure (22, 23). All procedures were performed in a standardised manner by a professional sonographer.

The pre-operative lower extremity Doppler ultrasonography was conducted by the same sonographer with a standard method. DVR was diagnosed when reflux was present and lasted longer than 1 s. For quantificationally judging the degree of DVR, we defined unilateral popliteal vein or superficial femoral vein (SFV) reflux as “1” point and unilateral common femoral vein (CFV) reflux as “2” points according to the anatomy of lower limb deep veins from calf to thigh. The final score for a patient was the summation for different types of DVRs.

### Age in Cardiac Doppler

According to previous studies (11, 24, 25), age is a significant factor influencing Doppler parameters for a healthy population. In our study, we analysed whether age had an effect on cardiac Doppler in the population with varicose veins to determine whether to conduct age matching when the *P*-value was <0.05 for inter-group age.

### Statistical Analysis

SPSS 26.0 version was used for statistical analysis. Doppler data for the control were presented as mean and standard deviation (SD) (Table 1). The continuous variables in our study were also expressed as mean and SD (Table 1). The unpaired *t*-test (Welch's—unequal variance) was used to analyse the differences between inter-group continuous variables (**Table 3**). Value of *p* < 0.05 was considered statistically significant.

**Table 1:** Cardiac Doppler parameters and features of primary varicose vein patients and the normal control.

**Parameter**	**Mean ± SD^a^**	**Mean ± SD^b^**
Age	55.2 ± 11.4	47.3 ± 16.0^*^
Number	129	1,394
Gender: female/male	74/55	716/678
**PW Doppler at the mitral valve**		
E wave velocity (cm/s)	73.4 ± 17.0	85.1 ± 20.0
A wave velocity (cm/s)	79.2 ± 22.9	69.6 ± 21.5
E/A ratio	1.02 ± 0.44	1.34 ± 0.49
**Tissue Doppler at the mitral valve**		
Septal e′ wave (cm/s)	8.0 ± 2.6	10.0 ± 3.1
Septal a′ wave (cm/s)	10.1 ± 2.3	9.1 ± 2.1
Septal s′ wave (cm/s)	9.0 ± 1.5	8.6 ± 1.7
Lateral e′ wave (cm/s)	11.5 ± 3.2	13.1 ± 4.0
Lateral a′ wave (cm/s)	12.3 ± 2.9	9.8 ± 2.7
Lateral s′ wave (cm/s)	11.4 ± 2.4	10.6 ± 2.6
Septal E/e′ ratio	9.8 ± 3.1	9.1 ± 3.0
Lateral E/e′ ratio	6.8 ± 1.9	7.0 ± 2.5
**PW Doppler at the tricuspid valve**		
E wave velocity (cm/s)	51.1 ± 10.6	57.5 ± 13.5
A wave velocity (cm/s)	46.9 ± 12.0	42.5 ± 11.5
E/A ratio	1.15 ± 0.33	1.45 ± 0.45
**Tissue Doppler at the tricuspid valve**		
e′ wave (cm/s)	10.4 ± 3.2	12.3 ± 3.5
a′ wave (cm/s)	15.3 ± 3.7	13.0 ± 3.8
s′ wave (cm/s)	13.4 ± 2.4	12.9 ± 2.5
E/e′ ratio	5.2 ± 1.6	5.0 ± 1.6

*PW, pulsed wave*.

a *Mean and standard deviation for patients with primary varicose veins*.

b *Mean and standard deviation for control derived from the EMINCA study (11)*.

* *p < 0.05, this difference is statistically significant*.

## Results

Valid information was available for 129 Chinese patients aged 23–80 years (mean age: 55.2 ± 11.4 years) with primary lower extremity varicose veins. Table 1 shows the cardiac Doppler parameters of patients with primary varicose veins and the normal control. The data of control were from the EMINCA study (11). Table 2 shows the influence of age on cardiac Doppler parameters in patients with varicose veins. There are only 90 patients who could be included to compare with control after age matching. Table 3 shows the difference in cardiac Doppler parameters between the 90 age-matched patients and normal control.

**Table 2:** The relationship between age and cardiac Doppler parameters in primary varicose vein patients.

**Parameters**	**20–40**	**40–60**	**60–80**	***p* ^a^**	***p* ^b^**	***p* ^c^**
Age	31.2 ± 5.1	51.2 ± 4.4	66.2 ± 4.7	0.0001^*^	0.0001^*^	0.0001^*^
Gender (M/F)	8/4	27/40	20/30	0.09	0.1	0.2
**PW Doppler** ^**a**^						
E wave (cm/s)	78.7 ± 17.9	73.0 ± 17.8	72.7 ± 15.6	0.3	0.3	0.5
A wave (cm/s)	48.5 ± 12.4	76.7 ± 15.9	89.9 ± 25.2	0.000^*^	0.000^*^	0.000^*^
E/A ratio	1.64 ± 0.26	0.99 ± 0.32	0.90 ± 0.49	0.000^*^	0.000^*^	0.000^*^
**Tissue Doppler** ^**a**^						
Septal e′ wave	12.3 ± 2.0	8.2 ± 2.3	6.7 ± 1.7	0.000^*^	0.000^*^	0.000^*^
Septal a′ wave	8.1 ± 1.0	10.4 ± 2.3	10.4 ± 2.3	0.000^*^	0.002^*^	0.003^*^
Septal s′ wave	9.4 ± 1.0	9.2 ± 1.4	8.6 ± 1.6	0.8	0.1	0.06
Lateral e′ wave	15.7 ± 3.3	11.6 ± 3.0	10.2 ± 2.5	0.000^*^	0.000^*^	0.000^*^
Lateral a′ wave	9.1 ± 1.9	12.5 ± 2.8	12.8 ± 2.8	0.000^*^	0.000^*^	0.000^*^
Lateral s′ wave	13.0 ± 2.3	11.8 ± 2.6	11.6 ± 1.8	0.1	0.06	0.001^*^
Septal E/e′ ratio	6.4 ± 1.2	9.3 ± 2.8	11.2 ± 2.9	0.000^*^	0.000^*^	0.000^*^
Lateral E/e′ ratio	5.2 ± 1.5	6.6 ± 2.0	7.4 ± 1.7	0.02^*^	0.000^*^	0.001^*^
**PW Doppler** ^**b**^						
E wave (cm/s)	56.5 ± 10.7	51.7 ± 10.2	48.9 ± 10.7	0.1	0.03^*^	0.02^*^
A wave (cm/s)	40.1 ± 8.3	45.9 ± 11.0	49.9 ± 13.2	0.09	0.02^*^	0.006^*^
E/A ratio	1.4 ± 0.20	1.18 ± 0.30	1.05 ± 0.35	0.01^*^	0.002^*^	0.0001^*^
**Tissue Doppler** ^**b**^						
e′ wave (cm/s)	14.4 ± 4.4	10.3 ± 3.0	9.6 ± 2.5	0.001^*^	0.0001^*^	0.0001^*^
a′ wave (cm/s)	11.2 ± 2.9	15.3 ± 3.6	16.3 ± 3.5	0.0001^*^	0.0001^*^	0.0001^*^
s′ wave (cm/s)	13.6 ± 1.4	13.0 ± 2.2	13.9 ± 2.8	0.4	0.8	0.3
E/e′ ratio	4.1 ± 1.1	5.3 ± 1.5	5.4 ± 1.7	0.01^*^	0.02^*^	0.04^*^

*p^a^Comparison of tricuspid Doppler between 40–60 age group and 20–40 age group*.

*^b^Comparison of tricuspid Doppler between 60–80 age group and 20–40 age group*.

*p^c^Variance analysis between different age groups and tricuspid Doppler*.

*PW Doppler^a^ and Tissue Doppler^a^: PW Doppler and Tissue Doppler at the mitral valve*.

*PW Doppler^b^ and Tissue Doppler^b^: PW Doppler and Tissue Doppler at the tricuspid valve*.

*M/F, Male/Female*.

* *p-value means p < 0.05, this difference is statistically significant*.

**Table 3:** Differences in cardiac Doppler parameters between age-matched patients and the normal control.

**Parameter**	**Mean ±SD**	**Mean difference with control^b^**	***P***
Age^m^ (*n*^a^)	49.7 ± 10.0 (90)	+2.4 (+5.1%)	0.16
Gender: female/male (*n*)	57%	+6%	0.33^**^
**PW Doppler at the mitral valve**			
E wave velocity (cm/s)	74.3 ± 17.7	−10.8 (−12.7%)	<0.0001^*^
A wave velocity (cm/s)	74.5 ± 20.9	+4.9 (+7.0%)	0.03^*^
E/A ratio	1.09 ± 0.47	−0.25 (−18.7%)	<0.0001^*^
**Tissue Doppler at the mitral valve**			
Septal e′ wave (cm/s)	8.7 ± 2.6	−1.3 (−13.0%)	<0.0001^*^
Septal a′ wave (cm/s)	10.1 ± 2.2	+1.0 (+11.0%)	<0.0001^*^
Septal s′ wave (cm/s)	9.1 ± 1.3	+0.5 (+5.8%)	0.006^*^
Lateral e′ wave (cm/s)	12.1 ± 3.3	−1.0 (−7.6%)	0.02^*^
Lateral a′ wave (cm/s)	12.1 ± 3.0	+2.3 (+23.5%)	<0.0001^*^
Lateral s′ wave (cm/s)	11.8 ± 2.5	+1.2 (+11.3%)	<0.0001^*^
Septal E/e′ ratio	9.1 ± 2.8	0 (0%)	1
Lateral E/e′ ratio	6.5 ± 1.9	−0.5 (−7.1%)	0.06
**PW Doppler at the tricuspid valve**			
E wave velocity (cm/s)	52.8 ± 10.1	−4.7 (−8.2%)	0.001^*^
A wave velocity (cm/s)	45.7 ± 11.5	+3.2 (+7.5%)	0.01^*^
E/A ratio	1.21 ± 0.31	−0.24 (−16.6%)	<0.0001^*^
**Tissue Doppler at the tricuspid valve**			
e′ wave (cm/s)	10.9 ± 3.4	−1.4 (−11.4%)	0.0002^*^
a′ wave (cm/s)	15.1 ± 4.0	+2.1 (+16.2%)	<0.0001^*^
s′ wave (cm/s)	13.2 ± 2.2	+0.3 (+2.3%)	0.27
E/e′ ratio	5.2 ± 1.6	+0.2 (+4.0%)	0.25

*PW, pulsed wave*.

a *Number of patients after age matching*.

b *The mean difference with control calculated as follows: patients mean minus control group mean. Results are presented as values and percentages. Control group means were 100%*.

m *Matching age with the control group*.

* *p < 0.05, this difference is statistically significant*;

** *Calculated by Chi-square test*.

### Influence of Age on Cardiac Doppler in Patients With Varicose Veins

Age is a significant factor influencing Doppler parameters in healthy adult populations (11, 24, 25). We divided the patients into groups of 20–40, 40–60, and 60–80 years old for statistical analysis to determine whether age has an influence on Doppler parameters in patients with primary varicose veins. We found that, with increasing age, Doppler parameters in patients with varicose veins had a decrease in early diastolic inflow and annular velocities (tricuspid E wave and mitral and tricuspid e′ wave), increase in late diastolic mitral and tricuspid inflow and annular velocities (A-wave and a′- wave), and decrease in mitral and tricuspid E/A ratios (*p* < 0.05; Table 2). This changed pattern is similar to the influence of age on cardiac Doppler in the healthy adult population (11, 20). In the following analysis, age matching was performed if the *P*-value was <0.05 for inter-group age. A study showed that the average tricuspid E/A ratio was equal to 1.6 in the healthy population at the age of thirty, decreasing by 0.1 for subsequent per decade (20). Based on this algorithm, in our study, tricuspid E/A ratios in any age group of patients (Table 2) were equal to the tricuspid E/A ratios in healthy adults who were 20 years older. For example, in the 40–60 age group of patients, the tricuspid E/A ratio was ≈1.2, identical to that in a 70-year-old healthy population. The same pattern was observed in the other two age groups.

### We Found That the Cardiac Doppler Parameters of Primary Varicose Vein Patients Differed From Those of Normal Control in Several Respects

Our results showed that patients with varicose veins had significantly lower early diastolic inflow velocities (mitral E wave −10.8 cm/s and tricuspid E wave −4.7 cm/s) and annular velocities (septal e′ wave −1.3 cm/s, lateral e′ wave −4.7 cm/s, and tricuspid e′ wave −1.4 cm/s), significantly higher late diastolic inflow (mitral A wave +4.9 cm/s and tricuspid A wave +3.4 cm/s) and annular velocities (septal a′ wave +1.0 cm/s, lateral a′ wave +2.3 cm/s and tricuspid a′ wave +2.1 cm/s), significantly higher mitral systolic annular velocities (septal s′ wave +0.5 cm/s and lateral s′ wave +1.2 cm/s), significantly lower mitral and tricuspid E/A ratio (respectively −0.25 and −0.24), and unaltered mitral and tricuspid E/e′ ratio compared to the normal control (Table 3).

### Relationship Between DVR and Cardiac Doppler Parameters

**Figure 2:**
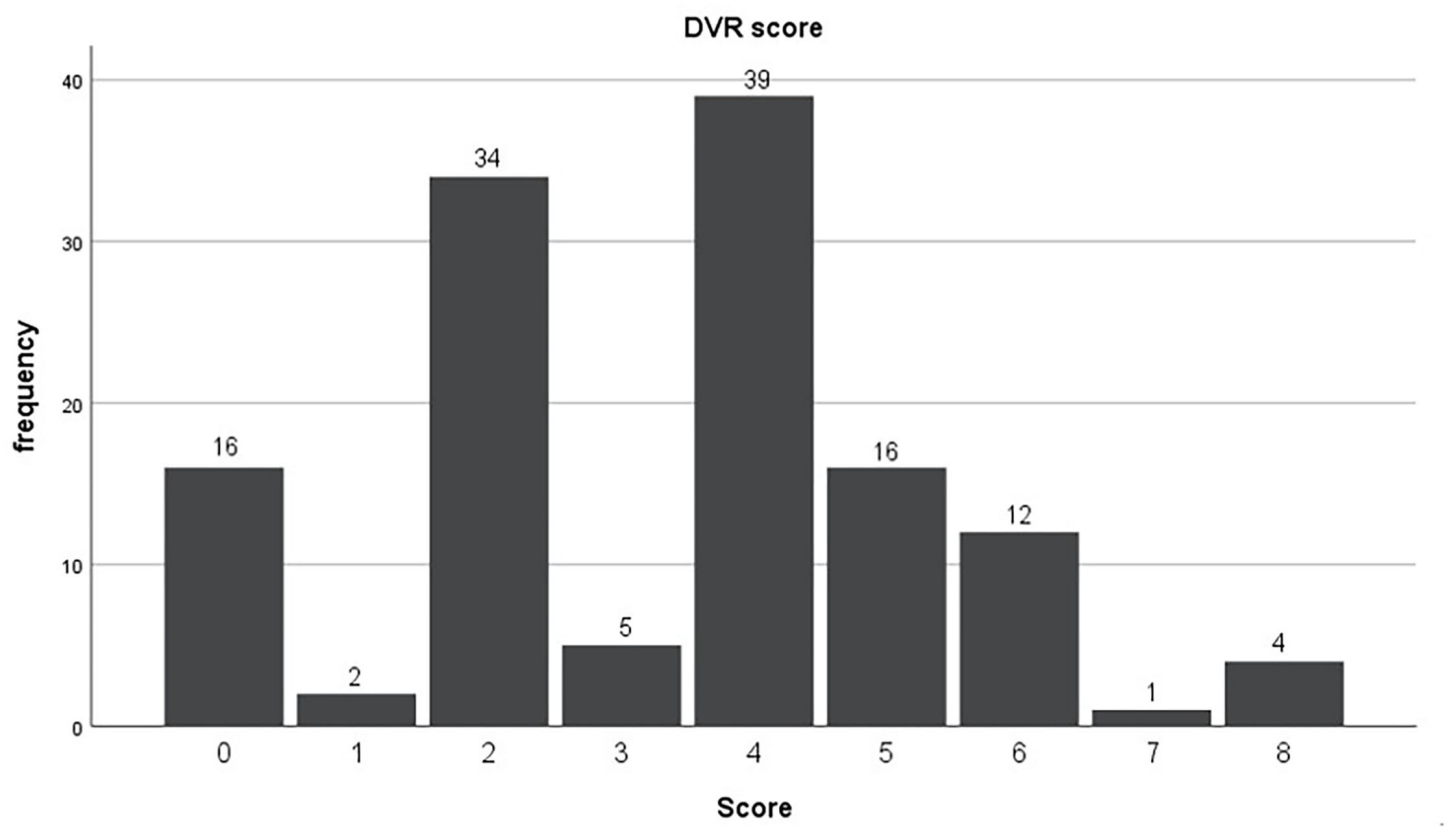
Deep venous reflux score.

According to the above DVR integral algorithm, all patients scored between 0 and 8 (Figure 2). First, we divided the non-DVR and DVR group according to whether there was DVR or not. The results showed that the *P*-values of almost all cardiac Doppler between the two groups were >0.05, indicating that the presence or absence of DVR had no statistically significant effect on cardiac Dopplers (Table 4). The *P* of septal E/e′ ratio was <0.05, but that alone was not enough to confirm if there was any difference in cardiac haemodynamics between non-DVR and DVR. Considering that there were only 16 patients in the non-DVR group, the limited sample size might lead to inter-group error. Next, we divided the patients by 3 points. Patients with scores ≤ 3 were classified to a mild DVR group (57 patients), while patients with scores ≥4 were classified to a severe DVR group (72 patients). The results showed that there was still no statistical difference between different degrees of DVR and cardiac Doppler parameters (Supplementary Table 4).

**Table 4:** Comparison of cardiac Doppler parameters between non-DVR and DVR patients.

	**Non-DVR**	**DVR**	***P***
Patients	16	113	
Age	58.8 ± 11.6	54.7 ± 11.4	0.2
**PW Doppler at the mitral valve**			
E wave velocity (cm/s)	78.7 ± 19.0	72.8 ± 16.6	0.2
A wave velocity (cm/s)	80.8 ± 19.9	79.3 ± 23.2	0.8
E/A ratio	1.05 ± 0.40	1.01 ± 0.45	0.8
**Tissue Doppler at the mitral valve**			
Septal e′ wave (cm/s)	7.0 ± 1.8	8.2 ± 2.6	0.09
Septal a′ wave (cm/s)	10.3 ± 2.2	10.1 ± 2.3	0.8
Septal s′ wave (cm/s)	8.7 ± 1.4	9.0 ± 1.5	0.4
Lateral e′ wave (cm/s)	10.6 ± 2.3	11.6 ± 3.3	0.3
Lateral a′ wave (cm/s)	12.0 ± 3.0	12.4 ± 2.9	0.6
Lateral s′ wave (cm/s)	10.9 ± 2.7	11.5 ± 2.4	0.4
Septal E/e′ ratio	11.8 ± 3.5	9.5 ± 2.9	0.006^*^
Lateral E/e′ ratio	7.6 ± 2.1	6.7 ± 1.9	0.06
**PW Doppler at the tricuspid valve**			
E wave velocity (cm/s)	54.3 ± 11.4	50.7 ± 10.5	0.2
A wave velocity (cm/s)	47.8 ± 10.6	46.9 ± 12.2	0.8
E/A ratio	1.17 ± 0.29	1.14 ± 0.34	0.8
**Tissue Doppler at the tricuspid valve**			
e′ wave (cm/s)	11.3 ± 3.1	10.3 ± 3.3	0.2
a′ wave (cm/s)	15.5 ± 3.5	15.3 ± 3.7	0.8
s′ wave (cm/s)	13.5 ± 1.8	13.4 ± 2.5	0.9
E/e′ ratio	5.0 ± 1.4	5.3 ± 1.6	0.5

*DVR, deep vein reflux; PW, pulsed wave*.

* *p < 0.05, this difference is statistically significant*.

### We Found That the Relationship Between the Cardiac Doppler Parameters and CEAP Clinical Class Were as Follows

The increase of CEAP clinical class indicated the progress of clinical manifestation. We, respectively, compared the difference of cardiac Doppler parameters between C2, C3, C4+C5+C6 class patients, and normal controls (Table 5). The results showed that C2 class patients basically had similar cardiac Dopplers with normal controls (*p* > 0.05). But when CEAP clinical class was equal to or greater than C3, cardiac Doppler parameters changed (*p* < 0.05) as follows: significantly lower early diastolic mitral, tricuspid inflow and annular velocities (E and e′ waves), significantly higher late diastolic mitral annular velocities (a′ waves), tricuspid inflow, and annular velocities (A and a′ waves), significantly higher mitral systolic annular velocities (s′ wave), significantly lower mitral and tricuspid E/A ratio, and normal mitral and tricuspid E/e′ ratio. Therefore, we found a potential CEAP grade cut-off value (C3) linked to statistical changes in cardiac Doppler parameters.

**Table 5:** Difference statistical analysis in cardiac Doppler parameters between different CEAP clinical class age-matched patients and normal control.

**Parameters**	**Control**	**C2**	**C3**	**C4+C5+C6**	**pC2**	**pC3**	**pC-h**
Age	47.3 ± 16.0	52.5 ± 12.7 (51)	55.3 ± 11.0 (28)	57.8 ± 9.8 (50)	0.02^*^	0.009^*^	0.000^*^
Age^m^	47.3 ± 16.0	48.9 ± 12.1 (38)	49.7 ± 9.1 (18)	49.7 ± 6.7 (23)	0.5	0.5	0.4
Gender (M/F)	678/716	15/23	7/11	15/8	0.3	0.4	0.1
**PW Doppler** ^**a**^							
E wave (cm/s)	85.1 ± 20.0	79.1 ± 21.6	73.8 ± 15.7	66.5 ± 10.9	0.1	0.007^*^	0.000^*^
A wave (cm/s)	69.6 ± 21.5	69.4 ± 22.2	76.6 ± 16.1	73.3 ± 19.3	0.9	0.08	0.4
E/A ratio	1.34 ± 0.49	1.26 ± 0.57	1.00 ± 0.31	0.99 ± 0.37	0.4	0.000^*^	0.000^*^
**Tissue Doppler** ^**a**^							
Septal e′ wave	10.0 ± 3.1	9.3 ± 2.8	8.2 ± 2.6	8.2 ± 2.2	0.1	0.01^*^	0.002^*^
Septal a′ wave	9.1 ± 2.1	9.4 ± 2.3	10.7 ± 1.7	10.7 ± 2.4	0.5	0.001^*^	0.005^*^
Septal s′ wave	8.6 ± 1.7	8.8 ± 1.2	9.2 ± 1.1	9.7 ± 1.5	0.4	0.03^*^	0.001^*^
Lateral e′ wave	13.1 ± 4.0	12.7 ± 3.2	11.4 ± 3.0	11.3 ± 3.9	0.4	0.03^*^	0.01^*^
Lateral a′ wave	9.8 ± 2.7	10.2 ± 2.4	13.2 ± 3.2	12.1 ± 3.0	0.2	0.000^*^	0.001^*^
Lateral s′ wave	10.6 ± 2.6	11.2 ± 2.5	11.7 ± 1.8	11.8 ± 2.5	0.1	0.02^*^	0.03^*^
Septal E/e′ ratio	9.1 ± 3.0	9.0 ± 2.8	9.6 ± 3.3	8.3 ± 2.3	0.9	0.5	0.1
Lateral E/e′ ratio	7.0 ± 2.5	6.5 ± 1.9	6.8 ± 2.0	6.3 ± 1.9	0.1	0.7	0.08
**PW Doppler** ^**b**^							
E wave (cm/s)	57.5 ± 13.5	56.2 ± 9.1	50.8 ± 11.7	50.2 ± 9.9	0.6	0.03^*^	0.01^*^
A wave (cm/s)	42.5 ± 11.5	40.7 ± 6.8	46.2 ± 9.9	47.5 ± 14.3	0.3	0.2	0.04^*^
E/A ratio	1.45 ± 0.45	1.40 ± 0.19	1.14 ± 0.32	1.14 ± 0.33	0.1	0.003^*^	0.001^*^
**Tissue Doppler** ^**b**^							
e′ wave (cm/s)	12.3 ± 3.5	11.8 ± 3.7	10.2 ± 2.2	10.6 ± 2.9	0.4	0.01^*^	0.02^*^
a′ wave (cm/s)	13.0 ± 3.8	13.9 ± 4.0	14.2 ± 3.7	15.1 ± 4.4	0.1	0.2	0.03^*^
s′ wave (cm/s)	12.9 ± 2.5	13.0 ± 1.8	12.8 ± 1.9	13.6 ± 2.5	0.8	0.9	0.2
E/e′ ratio	5.0 ± 1.6	5.1 ± 1.6	5.2 ± 1.6	5.1 ± 1.6	0.6	0.7	0.6

*CEAP, Clinical Etiological Anatomical Pathophysiological; PW, pulsed wave. C-h means C4+C5+C6. Age^m^: Post-matching age. M/F: Male/Female*.

*PW Doppler^a^ and Tissue Doppler^a^: PW Doppler and Tissue Doppler at the mitral valve*.

*PW Doppler^b^ and Tissue Doppler^b^: PW Doppler and Tissue Doppler at the tricuspid valve*.

*pC2: Comparison of age or Doppler parameters between C2 and normal control*.

*pC3: Comparison of age or Doppler parameters between C3 and normal control*.

*pC-h: Comparison of age or Doppler parameters between C4+C5+C6 and normal control*.

* *p means p-value < 0.05, this difference is statistically significant*.

## Discussion

For all we know, this is the inchoate study to evaluate the relationship between cardiac Doppler parameters and primary lower extremity varicose veins in the Chinese population.

### Cardiac Doppler Parameters and CEAP in Patients With Varicose Veins

We found some difference in cardiac Doppler parameters between primary varicose vein patients and the healthy Chinese population when there was no age difference. In addition, the changes in cardiac Doppler parameters were correlated with the CEAP clinical class and not with DVR status. In comparison with normal control, Doppler parameters of C2 clinical class patients were basically unchanged (*p* > 0.05). Whereas, the cardiac Doppler of the C3 or higher CEAP clinical class patients changed (*p* < 0.05) as follows: significantly lower early diastolic mitral, tricuspid inflow, and annular velocities (E and e′ waves), significantly higher late diastolic mitral annular velocities (a′ waves), tricuspid inflow, and annular velocities (A and a′ waves), significantly higher mitral systolic annular velocities (s′ wave), significantly lower mitral and tricuspid E/A ratio, and unaltered mitral and tricuspid E/e′ ratio. There are possible mechanisms underlying these cardiac haemodynamic changes. Firstly, non-functional venous valves and subsequent venous reflux in the superficial veins lead to visual varicose veins and peripheral venous blood stasis. In the blood circulation system, blood stasis would result in reducing venous return. In the cardiac Doppler parameters, we could see that decreasing early diastolic inflow E and annular e′ waves represent decreasing atrioventricular pressure gradient and pre-load (14, 15). To maintain end-diastolic ventricular volume, compensatory atrial contraction increases in the late diastolic stage (18, 19). In the Doppler, it is shown as increasing in late diastolic mitral, tricuspid inflow, and annular velocities (A and a′ waves). Our results revealed that C3 clinical class might be the cut-off correlated to cardiac Doppler parameter change in patients with primary varicose veins. Therefore, for C3 or higher CEAP clinical class patients, pre-operative echocardiography can be used to evaluate cardiac hemodynamic changes. However, large-scale clinical promotion requires further large sample studies.

It should be noted that this study was based on pre-operative patients, hence all results could only represent the pre-operative status of the patients. However, whether cardiac Doppler changes are simply caused by preoperative peripheral venous blood stasis or not. It is necessary to conduct a post-operative echocardiography examination for further analysis when peripheral venous blood stasis is relieved after the operation, particularly in patients whose CEAP grades are C3 or higher. At present, the collection and analysis of post-operative data are in progress.

### Age and Cardiac Doppler

Age is a significant factor influencing Doppler parameters in healthy adult populations (11, 24, 25). Middle-aged and older adults are at a higher risk for varicose veins (3). Our study demonstrated that the influence rule of age on cardiac Doppler parameters in patients with primary varicose veins was similar to that in the healthy adult population. In our study, tricuspid E/A ratios in any age group patients (Table 2) were equal to tricuspid E/A ratios in healthy adults who were 20 years older. To some extent, this also showed that some cardiac Doppler parameters in patients with varicose veins are different from those in the general population.

## Limitations

First, this study, although prospective, was a single-centre study. The number of patients available for data analysis was also limited. Patients in the 20–40-year age group were relatively few. This might be related to the lower incidence of varicose veins in younger patients (3). However, we adopted standardised data collection and reasonable statistical analysis to ensure authenticity and reliability. At present, we have not obtained all the post-operative echocardiographic data, so we cannot carry out the post-operative analysis, but this part of work will be completed in later stages. Another limitation was that pulse-wave and tissue Doppler measurements were performed in a swift manner due to the measuring nature of echocardiography. However, the procedures were operated by a professional sonographer with a standardised manner to ensure the accuracy of measurement.

## Conclusion

Cardiac Doppler parameters in Chinese patients with primary lower extremity varicose veins differ from those in the general population. There was no significant connexion between DVR and cardiac haemodynamics. In comparison with normal control, Doppler parameters of C2 class patients are basically unchanged. However, when the clinical manifestation progresses to grade 3 or above, the cardiac Doppler changes. Therefore, it could be considered that cardiac Doppler changes are positively correlated with the progress of clinical symptoms in patients with varicose veins. The C3 clinical class may be the cut-off linked to cardiac Doppler parameter change. Further post-operative echocardiography analysis is necessary to verify the underlying causes of cardiac Doppler changes in patients with varicose veins.

## Data Availability Statement

The raw data supporting the conclusions of this article will be made available by the authors, without undue reservation.

## Ethics Statement

Written informed consent was obtained from the individual(s) for the publication of any potentially identifiable images or data included in this article.

## Author Contributions

JZ performed the data collection, data analysis, and the writing of the manuscript. ZW contributed to study design and data collection. QF conducted some data analysis. YM performed study design and manuscript modification. HH contributed to data collection. All authors read and approved the final manuscript.

## Conflict of Interest

The authors declare that the research was conducted in the absence of any commercial or financial relationships that could be construed as a potential conflict of interest.

## Publisher's Note

All claims expressed in this article are solely those of the authors and do not necessarily represent those of their affiliated organizations, or those of the publisher, the editors and the reviewers. Any product that may be evaluated in this article, or claim that may be made by its manufacturer, is not guaranteed or endorsed by the publisher.
