# Supplementary material for: Cardiac Doppler Parameters and Progress in Clinical Manifestation of Primary Lower Extremity Varicose Veins: A Prospective Study in China
Source: Front Surg. 2022 Feb 28;9:791598. doi: 10.3389/fsurg.2022.791598 (PMC8918652; doi:10.3389/fsurg.2022.791598)
Supplement: Supplementary file 1 [file Table_1.DOCX]

**S Table 4.** Comparison of cardiac Doppler parameters between Mild-DVR and Severe-DVR patients

|  | Mild-DVR | Severe-DVR | P |
| --- | --- | --- | --- |
| number | 57 | 72 |  |
| age | 54.2±12.3 | 56.1±10.8 | 0.3 |
| PW Doppler at the mitral valve |  |  |  |
| E wave velocity (cm/s) | 73.8±17.0 | 73.3±17.0 | 0.9 |
| A wave velocity (cm/s) | 79.0±21.1 | 79.8±24.2 | 0.8 |
| E/A ratio | 1.04±0.51 | 1.00±0.38 | 0.6 |
| Tissue Doppler at the mitral valve |  |  |  |
| Septal e′ wave (cm/s) | 7.9±2.5 | 8.1±2.7 | 0.7 |
| Septal a′ wave (cm/s) | 10.6±2.2 | 9.8±2.3 | 0.06 |
| Septal s′ wave (cm/s) | 9.1±1.6 | 8.9±1.5 | 0.5 |
| Lateral e′ wave (cm/s) | 11.5±2.8 | 11.4±3.6 | 1.0 |
| Lateral a′ wave (cm/s) | 12.3±2.9 | 12.3±3.0 | 1.0 |
| Lateral s′ wave (cm/s) | 11.8±2.7 | 11.1±2.1 | 0.1 |
| Septal E/ e′ ratio | 10.0±3.4 | 9.7±2.8 | 0.5 |
| Lateral E/ e′ ratio | 6.8±2.0 | 6.8±1.9 | 1.0 |
| PW Doppler at the tricuspid valve |  |  |  |
| E wave velocity (cm/s) | 51.7±11.3 | 50.6±10.1 | 0.6 |
| A wave velocity (cm/s) | 47.3±10.8 | 46.7±12.9 | 0.8 |
| E/A ratio | 1.14±0.31 | 1.16±0.35 | 0.7 |
| Tissue Doppler at the tricuspid valve |  |  |  |
| e′ wave (cm/s) | 10.7±3.5 | 10.2±3.1 | 0.4 |
| a′ wave (cm/s) | 15.5±3.9 | 15.2±3.5 | 0.6 |
| s′ wave (cm/s) | 13.8±2.6 | 13.1±2.2 | 0.08 |
| E/ e′ ratio | 5.2±1.5 | 5.3±1.7 | 0.5 |

DVR: deep vein reflux

PW: pulsed wave
